# Validity of Inertial Sensors for Assessing Balance Kinematics and Mobility during Treadmill-Based Perturbation and Dance Training

**DOI:** 10.3390/s21093065

**Published:** 2021-04-28

**Authors:** Ernest Kwesi Ofori, Shuaijie Wang, Tanvi Bhatt

**Affiliations:** 1 Department of Physical Therapy, University of Illinois at Chicago, Chicago, IL 60612, USA; kwesiofori19@gmail.com (E.K.O.); wangshj1985@gmail.com (S.W.); 2 Division of Cardiovascular Medicine, Medical College of Wisconsin, Milwaukee, WI 53226, USA

**Keywords:** inertial sensors, perturbation training, dance

## Abstract

Inertial sensors (IS) enable the kinematic analysis of human motion with fewer logistical limitations than the silver standard optoelectronic motion capture (MOCAP) system. However, there are no data on the validity of IS for perturbation training and during the performance of dance. The aim of this present study was to determine the concurrent validity of IS in the analysis of kinematic data during slip and trip-like perturbations and during the performance of dance. Seven IS and the MOCAP system were simultaneously used to capture the reactive response and dance movements of fifteen healthy young participants (Age: 18–35 years). Bland Altman (BA) plots, root mean square errors (RMSE), Pearson’s correlation coefficients (R), and intraclass correlation coefficients (ICC) were used to compare kinematic variables of interest between the two systems for absolute equivalency and accuracy. Limits of agreements (LOA) of the BA plots ranged from −0.23 to 0.56 and −0.21 to 0.43 for slip and trip stability variables, respectively. The RMSE for slip and trip stabilities were from 0.11 to 0.20 and 0.11 to 0.16, respectively. For the joint mobility in dance, LOA varied from −6.98–18.54, while RMSE ranged from 1.90 to 13.06. Comparison of IS and optoelectronic MOCAP system for reactive balance and body segmental kinematics revealed that R varied from 0.59 to 0.81 and from 0.47 to 0.85 while ICC was from 0.50 to 0.72 and 0.45 to 0.84 respectively for slip–trip perturbations and dance. Results of moderate to high concurrent validity of IS and MOCAP systems. These results were consistent with results from similar studies. This suggests that IS are valid tools to quantitatively analyze reactive balance and mobility kinematics during slip–trip perturbation and the performance of dance at any location outside, including the laboratory, clinical and home settings.

## 1. Introduction

Postural instability and falls are common occurrences in older adults and stroke survivors resulting in numerous injuries and hospitalizations [1,2,3]. Voluntary and reactive balance control are mechanisms of resolving fall risks in all populations, and these mechanisms can be improved or enhanced via alternative training programs such as dance and perturbation training [4,5,6,7,8,9,10]. Fall prevention training in the form of dance and perturbation training has been extensively studied and the training effect on physical performance has been evaluated in the clinical and laboratory settings by use of predominantly clinical outcome measures such as Berg Balance Scale (BBS), Timed-Up and Go (TUG), Six-Minute Walk Test (6 MWT) among others [4,6,9,10]. However, the optoelectronic motion capture system (MOCAP) is a silver standard tool for motion analysis, which provides objective data on gait, joint mobility, balance, and fall risk in studies related to perturbation, gait, and, recently, dance training [4,5,11]. This implies that biomechanical analysis of balance control has been studied extensively in the laboratory setting with large equipment such as the MOCAP systems (e.g., VICON). Nonetheless, major limitations of the MOCAP systems (MS) are that of cumbersomeness, immobility, and restricted use outside of the laboratory settings [12]. However, to ensure seamless translation and assessment of the laboratory training effect to the clinical and home settings, it is imperative that these studies are replicated in the clinics and in the home or community where older adults live by the use of inertial sensors.

Inertial sensors (IS) provide a new direction of motion analysis for the smooth and effective evaluation of the comprehensive kinematics of human motion [13,14]. Inertial sensors, such as Xsens, are readily available, cost-effective, small, lightweight, and movable motion capture systems, which can provide similar kinematic data to the bulkier MOCAP system. Normally, IS possess tri-axial accelerometer, magnetometers, and gyroscopes, therefore enabling measurement of kinematic variables such as joint, segment angles and center of mass (COM) during human motion [15]. The attachment of IS for the evaluation of kinematic data can be useful in analyzing human motion in any environment both in and outside the laboratory settings. Thus, IS may play a key role in translating the effect of biomechanical research to the everyday activities of health and pathological populations. A few studies have evaluated the validity of IS for human motion with variable outcomes ranging from moderate to excellent agreements using variables from standard measurements [13,14]. Though IS have been used for analyzing some kinematic variables of self-generated motion during testing for clinical balance measures [16,17], no study to date has assessed the validity of IS for evaluating kinematic variables of proactive and reactive balance control during slip and trip perturbations, and dance movements.

Proper gait in terms of appropriate stepping strategy forms the basis of human locomotion and fall prevention due to slips and trips [18,19]. The extent of gait parameters such as step length provides major information on the proper walking function required for daily activities. For instance, a shorter step length may be a predisposition to frequent falls and injuries in healthy and pathological populations [20]. Additionally, slow walking speed may be indicative of gait impairment, and a predisposition to falls and injuries, predominantly in aging [21]. Alternatively, knee joint angle extrapolated from thigh angle may play a critical role in proper gait function [22,23], especially in toe clearance in health and pathological populations. Although many feasibility studies with IS have been conducted on overground walking [12,24], fewer studies have examined the validities of these sensors on stepping mechanics in fall prevention. Additionally, since gait, joint angle and proper stepping mechanics play critical roles in fall incidence, it is imperative to determine the validity of IS in measuring these biomechanical factors related to fall risk.

Reactive balance strategies elicited upon exposure to an external support surface perturbation are common mechanisms of determining fall risk. These strategies form the basis for implementing perturbation training programs, to reduce fall incidence in healthy and pathological populations [4,19]. These reactive balance assessments are assessed mainly using commercially available motorized treadmills include the extent of measures such as step length, and COM state, i.e., COM position and velocity usually in standing (Stance Perturbation Test) [19]. The above-mentioned kinematic measures form the foundation for the determination of postural stability and reactive balance control during perturbation training. With the ability to measure and generate COM as MOCAP, the use of the more portable and cost-effective IS over MOCAP for perturbation training is a better alternative for assessment of reactive balance control and fall risk, especially in the home or community setting. However, due to the lack of a concurrent validity study, IS have not been utilized to examine reactive balance control on the Stance Perturbation Test.

Additionally, dance as a multisensory modality has the ability to improve gait, balance, and reduce fall risk [25]. However, only few studies examining kinematics of dance have been conducted with mainly the MOCAP system in predominantly controlled laboratory settings [11,26]. Outcome measures such as COM state, joint angle from body segments, and postural sway have been examined in these studies. With dance being a social activity, the transfer of its gains from the laboratory to the clinics, homes, and community is critical for the holistic wellbeing of participants, and in adhering to the guidelines and recommendations by the Public Health Departments across the country [27]. To achieve this, IS could be employed in conducting studies in the clinic, home, or community settings to ensure proper evaluation of the effect of dancing on mobility and fall risk during the performance of activities of daily living (ADLs). Additionally, to advance the use of dance therapy, it will be imperative to determine the accuracy of IS for measuring kinematic measures during dance prior to its implementation in home or community-based settings. Moreover, the testing of the validity of IS during the stand perturbation test and dancing is to encompass the two domains of balance control (reactive and volitional). While the stepping response elicited on the Stance Perturbation Test assesses rapid and discrete movement responses (having a defined onset and offset) [28] and serves to measure reactive control, and dance movements including both serial (consisting of series of discrete tasks), continuous movements (tasks with no recognizable beginning and end) [28] serve to measure volitional control.

Thus, the aim of the present study was to determine the concurrent validity of IS in measuring kinematic variables of balance and mobility related to fall risk during treadmill-based perturbation (slip and trip) and dancing. Perturbations of varying intensities and dancing with variable song paces were provided to participants. Then, the variables of IS were compared to those from the MOCAP system (silver standard) for equivalence and accuracy. It was hypothesized that there would be reasonably good to excellent agreement of values of the variables analyzed in both systems.

## 2. Materials and Methods

### 2.1. Participants

Fifteen (15) young participants (18–35 years) without prior exposure to treadmill-based perturbation, and without any involvement in dance routines for at least 1 year (via prescreening interview) consented to participate in this study. Participants responded to a general health questionnaire to ascertain their health status. All participants with recent surgery or any musculoskeletal or cardiovascular conditions, which may impede successful participation in the study were excluded from the study. This laboratory-based study was approved by the institutional review board (IRB) of the University of Illinois at Chicago and conducted in the Cognitive Motor and Balance Rehabilitation Laboratory.

### 2.2. Instrumentation

Kinematic data were simultaneously obtained with an 8-camera MOCAP system (Motion Analysis, Santa Rosa, CA, USA) and with 7 inertial sensors (size: 47 × 30 × 13 mm; weight: 16 g; MTw Awinda Wireless 3DOF Motion Tracker, Software: MotionMonitor xGen version 3.3.9.0 Xsens Technologies, Enschede, Netherlands). The 8-camera optoelectronic MOCAP system was able to sense infra-red rays emitted by 29 reflective markers (Helen Hayes marker set), which were attached to various segments or joints of participants such as the sacrum, thighs, shanks, knee, and ankle joints to generate both position, segment, joint angles, and COM state. Concurrently, the 7 IS were attached to the sacral region, thighs, shanks, and middle toe of each foot. These sensors are infused with tri-axial accelerometers, magnetometer (±1.9 g), and gyroscopes (±2000°/s). As such, these sensors can capture the position, joint angles (estimated from segment angles), and COM state of human motion [15]. To generate output data, the IS are calibrated for misalignment and the manufacturer’s specifications show a static accuracy of ±1.5° and dynamic accuracy of ±2.25°. By this calibration, the IS are able to provide accurate orientation angle data (yaw ψ, pitch θ, and roll φ) through built-in integration of gyroscope, angular rate, and magnetic field vector estimations while addressing drift error by data filtering [13]. Real-time data from the IS unit are collected and automatically saved on a computer, with a sampling frequency of 100 Hz [29]. The MOCAP and the IS systems were time-synchronized to record data concurrently.

### 2.3. Data Collection and Processing

For concurrent validity, participants underwent 2 activities, which included treadmill-based stance perturbation tests (slips and trips) of varying intensities from levels 1 to 5, and dance movements with three songs of varying paces from slow to fast. For the treadmill perturbation, the displacement, velocity, and acceleration were the same, but with opposite directions for slip and trip. Across all levels, the acceleration was always constant (6 m/s^2^), while the displacement increased from 0.21 m for level 1 to 0.36 m for level 5, and their peak velocity increased from 0.31 m/s to 0.86 m/s.

### 2.4. Slip and Trip-Like Perturbations Protocol (Stance Perturbation Test)

Reactive balance was evaluated with the Stance Perturbation Test. For this task, which was performed on an ActiveStep^®^ Treadmill (Simbex, Lebanon, NH), participants experienced a series of slip-like forward and trip-like backward stance perturbations. The slip and trip perturbations provided to participants were of different intensities with 10–35% (average of 21.5%) increments in intensity (belt velocity) from the lowest (level 1) to the highest (level 5). The recovery foot is the one that undergoes a compensatory step post-perturbation while the perturbed foot is the one that undergoes slipping or tripping by sliding either forward or backward on the belt of the treadmill. Postural stability variables of interest were mainly stability and step length at touchdown (TD) of the unperturbed limb negotiating a compensatory or recovery due to perturbation. These variables for each system were then matched for equivalence and accuracy. Since stability is usually determined after the first reactive stepping foot touchdown during slip and trip perturbations, stabilities of the lower limb at touchdown were the only measures considered for analysis. Thus, the outcome variables of interest for slip and trip perturbation validity test were stabilities at TD relative to the recovery foot and perturbed foot and step lengths at TD (step length_TD) of the recovery foot.

### 2.5. Dance Protocol

For this activity, participants dance to three songs of slow (120 beats per minute, bpm), medium (130 bpm), and fast pace (138 bpm). A protocol from a previous study [11] was implemented to deliver songs or dance steps. The only variation with this current protocol was that the three-trial recordings for each dance were set at a lesser time of 10 s in this study compared to 30 s in the referred study. The mean values of variables for the three trials were used for analysis. For analysis, segment angle excursions (difference between the maximum and minimum angle peaks) of the right and left thighs (Rt and Lt thighs), right and left shanks (Rt and Lt shanks), and the right and left feet (Rt and Lt feet) joints in the sagittal plane were compared between the two motion capture systems for equivalence and accuracy. The thigh and shank angles were determined by the calculation of the angle between the body segment and the vertical axes, while the foot segmental angles were established by calculating the angle between the foot and the horizontal axis.

### 2.6. Data Analysis

Data collected were processed by digitization via MOCAP and IS software to generate initial COM, gait, and segment angle data. Then, further processing was completed using a customized MATLAB code to generate COM state, step length of the recovery foot touchdown post-perturbation, and segment angles of the lower limbs. These data were then used to calculate stability values during the perturbation test.

To ensure that the coordinate systems were the same for the two systems, the calibration procedure was first used to determine the coordinated systems of IS and MOCAP systems. For the MOCAP calibration, an L-Frame was placed on the ground, the long arm determined the x-axis (or AP direction), the short arm determined the y-axis (or mediolateral, ML direction), the line vertical to both arms was the z-axis. For the IS, the north-south direction always represented the x-axis while the east-west direction represented the y-axis. To ensure both coordinate systems are identical, we placed the long arm of the L-Frame in the north-south direction and the short arm in the east–west direction for MOCAP calibration.

The COM state was calculated relative to the edge of BOS in the anterior–posterior (AP) direction, and then normalized by foot length or $\sqrt{g\times body\;height}$. The stability values were computed with a previously developed method of determining COM state. For the MOCAP system, the coordination of COM was computed from the kinematic data using known segmental parameter information in a 13-segment representation of the body. The edge of the BOS was represented by the heel of the perturbed foot and their velocity was calculated by taking the time derivative of the corresponding trajectory. While for the IS system, the COM position was estimated from the distance between the hip joint and the ankle joint of the perturbed limb in the AP direction using segmental length and angle, and the COM velocity was estimated using segment length, angle, and angular velocity. Similarly, the step length was calculated as the distance between the trailing heel and leading heel for the MOCAP system, and for IS system, it was calculated as the distance between ankle joints using segment length and angle. For IS, segment angles were exported directly from the IS processing system, but for the MOCAP system, the segment angles were calculated using joint position data. The primary outcome measures (segment angle excursions) for dance, were computed using segmental method formulas which were proposed [30] and implemented in a previous study [11].

### 2.7. Statistics

The Kolmogorov–Smirnov test was used to check for normality of data with a paired *t*-test performed to determine any significant differences between variables of IS and MOCAP systems. A concurrent validity analysis via SPSS (IBM Corporation, Endicott, NY) was conducted to determine the similarity between the outcome variables for the two systems during the performance of each task. Initially, Bland Altman (BA) plots and the determination of root mean square error (RMSE) were used to ascertain the agreements of stability and mobility variables between the two motion capture systems. Secondly, correlation coefficient (R) and intra-class correlation (ICC) were used to determine the agreement of variables between the IS and MOCAP systems. To demonstrate the effect of the song on body segment, correlations between each song pace and each segmental angle excursion were determined. The significant level of *p*-values, α, was set at 0.05.

## 3. Results

All data were found to be normally distributed.

### 3.1. Slip and Trip-Like Perturbations (Stance Perturbation Test)

**Figure 1:**
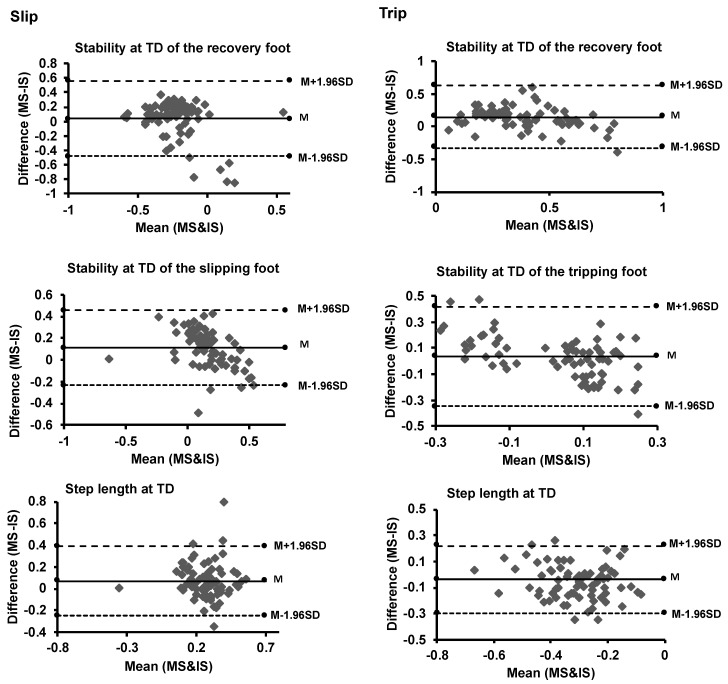
Bland-Altman plots of postural stability measures (of the Stance Perturbation Test) for motion (MS) and inertial sensor (IS) systems. The solid line represents the mean (M), while the two dash lines above and below M represent the upper (M + 1.96 SD) and lower (M − 1.96 SD) limits of agreements (1.96 standard deviations), respectively.

For slip perturbations, analysis conducted with 75 data points from 15 subjects included data of five slip–trip levels or intensities from each participant. For all reactive balance and stability variables, paired *t*-tests showed no significant differences between the two systems (*p* > 0.05). Validity analysis of reactive balance variables during treadmill-based slip and trip perturbations depicted moderate to high level of similarity of values obtained from the MOCAP and IS systems. This was demonstrated by the Bland Altman plots, Limits of Agreements and the calculation of RMSE, shown in Table 1, (Figure 1). Additionally, the R and CC of stability and mobility variables of slips and trips shown in Table 1 depicted significant positive correlations and similarities between the two systems MOCAP and IS (*p* < 0.05).

### 3.2. Dance

**Figure 2:**
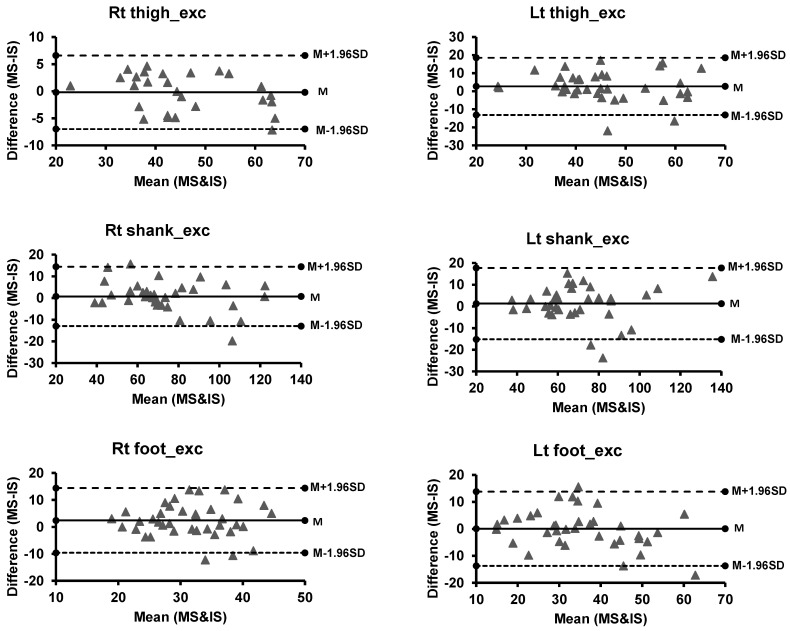
Bland-Altman plots of segment mobility measures (Dance test) for motion (MS) and inertial sensor (IS) systems. The solid line represents the mean (M), while the two dash lines above and below M represent the upper (M + 1.96 SD) and lower (M − 1.96 SD) limits of agreements (1.96 standard deviations), respectively. Abbreviation: Lt = Left, Rt = Right, exc = excursion.

For the dance task, there were 42 data points obtained from 14 participants, which consisted of data for three dance songs from each participant. Data for one participant were excluded because of noise in data created by distortions in the IS system. Results conducted by paired t-tests for mobility variables during the dance movements with the three paces of the song showed no significant differences of variables between the two motion capture systems (*p* > 0.05). Bland Altman plots, Limits of Agreements and RMSE are shown in Table 2 (see Figure 2). Additionally, R and ICC scores depicted moderate to high validity values for all segment angle excursions (*p* < 0.05). The R, and ICC scores for mobility variables included segmental angle excursions are shown in Table 2.

For each song, there was good concurrent validity of joint mobility values between the two motion capture systems because the R and ICC positively correlated with all segment angles. Dancing with slow-paced song correlated highly with Rt shank and Lt foot angle excursions (*p* < 0.05). With the medium-paced song, there were significant correlations with Lt shank and foot excursions, while dance the fast-paced song demonstrated significant correlations with all segmental angle excursions (*p* < 0.05), except for the Lt foot excursion, see Table 3.

## 4. Discussion

This study demonstrated that motion analysis via inertial sensors could provide precise kinematic data on body segments equivalent to the optoelectronic MOCAP system during slip and trip-like perturbations (Stance Perturbation Test was used to evaluated reactive balance), and in dance performance. In this study, the reactive balance and mobility measures (stability and step length at touchdown) during slip and trip perturbations were highly comparable in both systems. Equally, the mobility measures (segment angle excursions) during dancing were highly equivalent in both systems. This implies that the hypothesis of similarity and reasonably good agreement (with relatively close to 95% LOA) of values analyzed between both systems was adequately realized in this study. It should be noted that by advanced processing, joint angles could be extrapolated from the segment angles (raw data extracted from both systems) presented in the results.

The optoelectronic MOCAP system, as a silver standard tool for motion analysis, has been the most reliable tool predominantly utilized in clinical studies related to reactive balance control in varying populations in the laboratory setting [4,19,31]. Albeit, this system comes along with logistical constraints and thus results in potential minor measurement errors from soft tissue and skin artifacts stemming from the shifting of reflective markers [13]. Inertial sensors provide the added advantage over the MOCAP system of being portable and therefore can be utilized both in the laboratory, clinic, home, and community settings for assessment of health outcomes through data collection, processing, and analysis via system-specific software [13,14,32].

Laboratory-based studies on fall prevention have examined reactive balance control during slip and trip perturbations with key variables such as stability and joint angles at touchdown [19,33]. These above-mentioned variables are major determinants of fall risk in aging, and in neurologically-impaired individuals [34]. Generally, our study depicted moderate to high R and ICC values for all variables during the perturbations. This implies moderate to high agreement and accuracy of outcomes of reactive balance control and mobility between the two systems. It also signifies moderate to high validity of data obtained from IS for the analysis of reactive balance control. The results of the present study are in accordance with other studies, which showed moderate to the excellent similarity of gait, balance, and mobility kinematic variables between the two systems [13,35,36,37,38]. The results of this study would provide more confidence in conducting studies related to reactive balance control during perturbation training, and assess the training effectiveness in the laboratory and, more importantly, in the home and community settings by the application of IS.

Dance is an emerging alternative intervention that is generally accepted and enjoyed by every population group and is increasingly being implemented to improve gait, balance, and mobility or range of motion [26,39]. Additionally, dance has been extensively studied in the young and aging populations to depict improvement in clinical balance measures such as Berg Balance Scale (BBS) and Timed Up and Go (TUG) [26,39,40,41,42], but these measures are predominantly insensitive and possess floor effect [17]. Therefore, the clinical gains of dance may not translate into real-life situations. As such, the MOCAP system was resorted to in the evaluation of the efficacy of dance by the provision of objective data of balance and mobility [43]. However, with dance being a social event enjoyed by all ages, there is the need to evaluate its effectiveness on balance and mobility with a portable, lightweight IS system, which provides similar variables as the silver standard optoelectronic MOCAP system [13]. The report of segment angle excursions in this study is vital because they provide an estimate of the extent of joint movement during the performance of dance with varying paces of songs. Our study also revealed moderate to high R and ICC as the absolute agreements and accuracy of segment angle excursions were compared between IS and MOCAP systems. This shows that generally, there was moderate to high validity of IS in the evaluation of segment angle excursions during the performance of dance with varying paces of song. It also demonstrated that IS may be a great tool for assessing segment angle excursions during the performance of dance. With dance being cyclical and depicting similar attributes to gait, studies on gait and other daily activities revealed high validity of IS in analysis with joint angles [13,36,37] and high agreement of variable values between IS and MOCAP systems [36,37]. These results could provide increased assurance in the kinematic analysis of dance movement using IS. The IS system could thus serve as the ideal tool for data collection, analysis, and the evaluation of the effectiveness of dance therapy implemented in all settings, including the research laboratory, clinic, home, or community settings to show improvements in health outcomes and advance the course of a home or community-based therapy.

## 5. Conclusions

This study demonstrated the concurrent validity of IS by showing that analysis of reactive balance and mobility outcome measures during slip and trip perturbations assessed via the Stance Perturbation Test could be performed with IS with high accuracies, like the MOCAP systems. Though there is scanty evidence on studies on dance training, which have been conducted with MOCAP system, this study showed that studies on dance therapy could be also conducted to determine its effects on postural control and mobility measures with IS. With the determination of the moderate to high validity of IS for assessing balance kinematics during slip and trip-like perturbations and dancing, there is increased assurance for clinicians or researchers to evaluate the progress of home therapy interventions. Additionally, with the current momentum shifting towards the translation of the benefits of research into the daily activities of healthy aging, and neurological-impaired individuals such as stroke, the increased validity of IS will pave the way for the increased home or community-based research and employ the portable IS tool to conduct an objective assessment of balance and fall risk of participants during perturbation training and dance training.

## Figures and Tables

**Table 1:** Validity data of postural stability variables for slip–trip perturbation tests.

Variable	R Value	ICC	RMSE	Limits of Agreement
**SLIP**				
Stability at TD of the recovery foot	0.59	0.56 *	0.20	−0.48–0.56
Stability at TD of the slipping foot	0.81	0.72 *	0.17	−0.23–0.45
Step length at TD	0.68	0.64 *	0.11	−0.24–0.39
**TRIP**				
Stability at TD of the recovery foot	0.61	0.50 *	0.16	−0.21–0.43
Stability at TD of the slipping foot	0.77	0.71 *	0.15	−035–0.42
Step length at TD	0.68	0.64 *	0.11	−0.29–0.22

Abbreviations: TD = Touchdown, R = correlation coefficient, ICC = intraclass correlation coefficient, RMSE = root mean square error, significant level of validity was shown by * denoting *p* < 0.05.

**Table 2:** Validity of segment angle excursions (exc) during dance testing.

Variable	R Value	ICC	RMSE	Limits of Agreement
Rt thigh_exc	0.71	0.71 *	1.9	−6.98–6.60
Rt shank_exc	0.78	0.77 *	12.46	−12.94–14.43
Rt foot_exc	0.47	0.45 *	7.69	−9.60–14.41
Lt thigh_exc	0.60	0.59 *	9.79	−13.11–18.54
Lt shank_exc	0.85	0.84 *	13.06	−15.18–17.72
Lt foot_exc	0.79	0.71 *	9.28	13.72–13.81

Significant level of validity was shown by * denoting *p* < 0.05.

**Table 3:** Correlations between each song pace and segment angle excursions.

	Slow-Paced Song			Medium-Paced Song			Fast-Paced Song		
	R	ICC	Cronbach’s Alpha	R	ICC	Cronbach’s Alpha	R	ICC	Cronbach’s Alpha
Rt thigh_exc	0.28	0.38	0.44	0.38	0.53	0.55	0.77 *	0.86	0.87
Rt shank_exc	0.80 *	0.87	0.89	0.45	0.54	0.60	0.75 *	0.72	0.72
Rt foot_exc	0.12	0.14	0.13	0.38	0.51	0.55	0.57 *	0.67	0.67
Lt thigh_exc	0.41	0.41	0.52	0.25	0.36	0.39	0.53 *	0.67	0.68
Lt shank_exc	0.35	0.47	0.52	0.79 *	0.82	0.88	0.82 *	0.58	0.86
Lt foot_exc	0.87 *	0.93	0.93	0.73 *	0.80	0.81	0.4	0.32	0.33

Significant level of correlation (R) was shown by * denoting *p* < 0.05. Abbreviation: Lt = Left, Rt = Right, exc = excursion.

## Data Availability

The data presented in this study are available on request from the corresponding author. The data are not publicly available due to privacy issue.

## References

[1] Alexander B.H., Rivara F.P., Wolf M.E. (1992). The cost and frequency of hospitalization for fall-related injuries in older adults. Am. J. Public Health.

[2] King M.B., Tinetti M.E. (1995). Falls in community-dwelling older persons. J. Am. Geriatrics Soc..

[3] Yates J.S., Lai S.M., Duncan P.W., Studenski S. (2002). Falls in community-dwelling stroke survivors: An accumulated impairments model. J. Rehabil. Res. Dev..

[4] Pai Y.-C., Bhatt T., Yang F., Wang E., Kritchevsky S. (2014). Perturbation training can reduce community-dwelling older adults’ annual fall risk: A randomized controlled trial. J. Gerontol. Ser. A Biomed. Sci. Med. Sci..

[5] Parijat P., Lockhart T.E. (2012). Effects of moveable platform training in preventing slip-induced falls in older adults. Ann. Biomed. Eng..

[6] Fitzgerald G.K., Childs J.D., Ridge T.M., Irrgang J.J. (2002). Agility and perturbation training for a physically active individual with knee osteoarthritis. Phys. Ther..

[7] Granacher U., Muehlbauer T., Bridenbaugh S.A., Wolf M., Roth R., Gschwind Y., Wolf I., Mata R., Kressig R.W. (2012). Effects of a salsa dance training on balance and strength performance in older adults. Gerontology.

[8] Sofianidis G., Hatzitaki V., Douka S., Grouios G. (2009). Effect of a 10-week traditional dance program on static and dynamic balance control in elderly adults. J. Aging Phys. Act..

[9] Eyigor S., Karapolat H., Durmaz B., Ibisoglu U., Cakir S. (2009). A randomized controlled trial of Turkish folklore dance on the physical performance, balance, depression and quality of life in older women. Arch. Gerontol. Geriatr..

[10] McKinley P., Jacobson A., Leroux A., Bednarczyk V., Rossignol M., Fung J. (2008). Effect of a community-based Argentine tango dance program on functional balance and confidence in older adults. J. Aging Phys. Act..

[11] Ofori E.K., Subramaniam S., Wang S., Bhatt T. (2019). Kinematic analysis of dance-based exergaming: Effect of song pace on center of mass and joint mobility. J. Phys. Ther. Sci..

[12] Saber-Sheikh K., Bryant E.C., Glazzard C., Hamel A., Lee R.Y. (2010). Feasibility of using inertial sensors to assess human movement. Man. Ther..

[13] Bolink S., Naisas H., Senden R., Essers H., Heyligers I., Meijer K., Grimm B. (2016). Validity of an inertial measurement unit to assess pelvic orientation angles during gait, sit–stand transfers and step-up transfers: Comparison with an optoelectronic motion capture system. Med. Eng. Phys..

[14] Donath L., Faude O., Lichtenstein E., Nüesch C., Mündermann A. (2016). Validity and reliability of a portable gait analysis system for measuring spatiotemporal gait characteristics: Comparison to an instrumented treadmill. J. Neuroeng. Rehabil..

[15] Gouwanda D., Gopalai A.A. (2015). A robust real-time gait event detection using wireless gyroscope and its application on normal and altered gaits. Med. Eng. Phys..

[16] Coulthard J.T., Treen T.T., Oates A.R., Lanovaz J.L. (2015). Evaluation of an inertial sensor system for analysis of timed-up-and-go under dual-task demands. Gait Posture.

[17] Mancini M., Horak F.B. (2010). The relevance of clinical balance assessment tools to differentiate balance deficits. Eur. J. Phys. Rehabil. Med..

[18] Kovacs C.R. (2005). Age-related changes in gait and obstacle avoidance capabilities in older adults: A review. J. Appl. Gerontol..

[19] Patel P., Bhatt T. (2015). Adaptation to large-magnitude treadmill-based perturbations: Improvements in reactive balance response. Physiol. Rep..

[20] Verghese J., Holtzer R., Lipton R.B., Wang C. (2009). Quantitative gait markers and incident fall risk in older adults. J. Gerontol. Ser. A.

[21] Senden R., Savelberg H., Grimm B., Heyligers I., Meijer K. (2012). Accelerometry-based gait analysis, an additional objective approach to screen subjects at risk for falling. Gait Posture.

[22] Begg R., Sparrow W. (2006). Ageing effects on knee and ankle joint angles at key events and phases of the gait cycle. J. Med. Eng. Technol..

[23] Mills P.M., Barrett R.S., Morrison S. (2008). Toe clearance variability during walking in young and elderly men. Gait Posture.

[24] Mathie M.J., Coster A.C., Lovell N.H., Celler B.G. (2004). Accelerometry: Providing an integrated, practical method for long-term, ambulatory monitoring of human movement. Physiol. Meas..

[25] Alpert P.T., Miller S.K., Wallmann H., Havey R., Cross C., Chevalia T., Gillis C.B., Kodandapari K. (2009). The effect of modified jazz dance on balance, cognition, and mood in older adults. J. Am. Acad. Nurse Pract..

[26] Subramaniam S., Bhatt T. (2019). Dance-based exergaming for upper extremity rehabilitation and reducing fall-risk in community-dwelling individuals with chronic stroke. A preliminary study. Top. Stroke Rehabil..

[27] Brownson R.C., Kreuter M.W., Arrington B.A., True W.R. (2006). From the schools of public health. Public Health Rep..

[28] Schmidt R. (1988). Motor Control and Learning: A Behavioral Emphasis.

[29] Manual M.U. User Guide MVN, MVN BIOMECH MVN Link, MVN Awinda. **2019**, *1*, 33. https://www.xsens.com/hubfs/Downloads/usermanual/MVN_User_Manual.pdf.

[30] Prieto T.E., Myklebust J.B., Hoffmann R.G., Lovett E.G., Myklebust B.M. (1996). Measures of postural steadiness: Differences between healthy young and elderly adults. IEEE Trans. Biomed. Eng..

[31] Bhatt T., Espy D., Yang F., Pai Y.-C. (2011). Dynamic gait stability, clinical correlates, and prognosis of falls among community-dwelling older adults. Arch. Phys. Med. Rehabil..

[32] Agner S., Bernet J., Brülhart Y., Radlinger L., Rogan S. (2015). Spatiotemporal gait parameters during dual task walking in need of care elderly and young adults. Z. Gerontol. Geriatr..

[33] Salot P., Patel P., Bhatt T. (2016). Reactive balance in individuals with chronic stroke: Biomechanical factors related to perturbation-induced backward falling. Phys. Ther..

[34] Patel P.J., Bhatt T. (2016). Does aging with a cortical lesion increase fall-risk: Examining effect of age versus stroke on intensity modulation of reactive balance responses from slip-like perturbations. Neuroscience.

[35] Fong D.T.-P., Chan Y.-Y. (2010). The use of wearable inertial motion sensors in human lower limb biomechanics studies: A systematic review. Sensors.

[36] Buganè F., Benedetti M.G., D’Angeli V., Leardini A. (2014). Estimation of pelvis kinematics in level walking based on a single inertial sensor positioned close to the sacrum: Validation on healthy subjects with stereophotogrammetric system. Biomed. Eng. Online.

[37] Takeda R., Tadano S., Natorigawa A., Todoh M., Yoshinari S. (2009). Gait posture estimation using wearable acceleration and gyro sensors. J. Biomech..

[38] Papi E., Osei-Kuffour D., Chen Y.-M.A., McGregor A.H. (2015). Use of wearable technology for performance assessment: A validation study. Med. Eng. Phys..

[39] Subramaniam S., Bhatt T. (2015). Does a virtual reality-based dance training paradigm increase balance control in chronic stroke survivors? A preliminary study. Int. J. Neurorehabilit..

[40] Smith S.T., Sherrington C., Studenski S., Schoene D., Lord S.R. (2011). A novel Dance Dance Revolution (DDR) system for in-home training of stepping ability: Basic parameters of system use by older adults. Br. J. Sports Med..

[41] Hackney M.E., Hall C.D., Echt K.V., Wolf S.L. (2012). Application of adapted tango as therapeutic intervention for patients with chronic stroke. J. Geriatr. Phys. Ther..

[42] Hackney M.E., Earhart G.M. (2009). Effects of dance on movement control in Parkinson’s disease: A comparison of Argentine tango and American ballroom. J. Rehabil. Med..

[43] Muyor J.M., Arrabal-Campos F.M., Martinez-Aparicio C., Sanchez-Crespo A., Villa-Perez M. (2017). Test-retest reliability and validity of a motion capture (MOCAP) system for measuring thoracic and lumbar spinal curvatures and sacral inclination in the sagittal plane. J. Back Musculoskelet. Rehabil..

